# The possible protective effects of dipyridamole on ischemic reperfusion injury of priapism

**DOI:** 10.1590/S1677-5538.IBJU.2015.0072

**Published:** 2016

**Authors:** Ersagun Karaguzel, Cemil Bayraktar, Omer Kutlu, Esin Yulug, Ahmet Mentese, Ali Ertan Okatan, Fatih Colak, Serap Ozer, Ilke O.Kazaz

**Affiliations:** 1 Department of Urology, Faculty of Medicine, Karadeniz Technical University, Trabzon, Turkey;; 2 Department of Histology and Embryology, Karadeniz Technical University, Faculty of Medicine, Trabzon, Turkey;; 3 Department of Medical Biochemistry, Karadeniz Technical University, Faculty of Medicine, Trabzon, Turkey

**Keywords:** Priapism, Reperfusion Injury, Ischemia, Dipyridamole

## Abstract

**Purpose:**

To investigate the protective effects against ischemia reperfusion injury of dipyridamole in a model of induced priapism in rats.

**Materials and Methods:**

Twenty-four male Sprague-Dawley rats were divided into four groups, control, P/R, P/R+DMSO and P/R+D. 3ml blood specimens were collected from vena cava inferior in order to determine serum MDA, IMA, TAS, TOS and OSI values, and penile tissue was taken for histopathological examination in control group. Priapism was induced in P/R group. After 1h, priapism was concluded and 30 min reperfusion was performed. In P/R+DMSO group 1ml/kg DMSO was administered intraperitoneally 30 min before reperfusion, while in P/R+D group 10mg/kg dipyridamole was administered intraperitoneally 30 min before reperfusion. Blood and penis specimens were collected after the end of 30 min reperfusion period. Sinusoidal area (µm^2^), tears in tunica albuginea and injury parameters in sinusoidal endothelium of penis were investigated.

**Results:**

Histopathological examination revealed no significant changes in term of sinusoidal area. A decrease in tears was observed in P/R+D group compared to P/R group (p<0.05). Endothelial injury decreased in P/R+D group compared to P/R group (p>0.05). There were no significant differences in MDA and IMA values between groups. A significant increase in TOS and OSI values was observed in P/R+D group compared to P/R group. A significant decrease in TAS levels was observed in P/R+D group compared to the P/R group.

**Conclusions:**

The administration of dipyridamole before reperfusion in ischemic priapism model has a potential protective effect against histopathological injury of the penis.

## INTRODUCTION

Priapism is defined as complete or partial penile tumescence exceeding 4 h in length (1, 2). There are three types of priapism, ischemic (low flow, veno-occlusive), non-ischemic (high flow, arterial) and stuttering (repeating). Ischemic priapism is the most common form and represents a urological emergency (3). In low-flow priapism, a reduced venous flow in cavernous tissues, hypoxia related to venous stasis and histopathological changes associated with acidosis are observed. Irreversible corporal tissue necrosis and fibrotic tissue formation may occur if ischemia exceeds 4-6 h (4, 5).

Ischemia-reperfusion (I/R) injury occurs with termination of priapism (6). Although reperfusion is a necessary mechanism for the protection of ischemic tissue, reperfusion itself initiates a pathophysiological process that leads to injury. With I/R injury neutrophils, inflammatory cytokines and adhesion molecules with increased thrombogenicity are activated, and free oxygen radicals develop with the release of massive intracellular Ca (7, 8). Oxidative stress damages the cell membrane and has damaging effects on tissue by leading to permanent cellular injury.

Dipyridamole is a platelet inhibitor with antithrombocytic effects. It is a cyclic guanosine monophosphate (cGMP)-dependent phosphodiesterase and adenosine carrier inhibitor with antioxidant properties that prevents the formation of reactive oxygen radicals in endothelial cells and thrombocytes (9, 10). In addition to antiaggregant effects, it also possesses anti-inflammatory and neuroprotective effects. Experimental studies have shown that dipyridamole reduces I/R injury in various organs and tissues, such as the testis, liver and myocardium (11-13).

The purpose of this study was to determine the protective effects of dipyridamole against I/R injury in an induced experimental model of acute ischemic priapism in rats.

## MATERIALS AND METHODS

All animal experiments were performed following Karadeniz Technical University Animal Care and Ethics Committee approval, in compliance with the principles of laboratory animal care (National Institutes of Health publication no. 85-23, revised 1985). Twenty-four adult (approximately 4 months) male Sprague Dawley rats weighing 400-450 gr were used in the study. Rats were placed in cages 1 week before the study for adaptation and given standard rat chow and tap water. Rats were numbered and randomized by lots. Following randomization, rats were divided into four groups of six rats each (n=6). Throughout the experiment all animals were kept under standard room temperature (23±2ºC), lighting (12 h light/12 h dark) and humidity conditions (50%±10).

### Experimental design and groups

**Figure 1:**
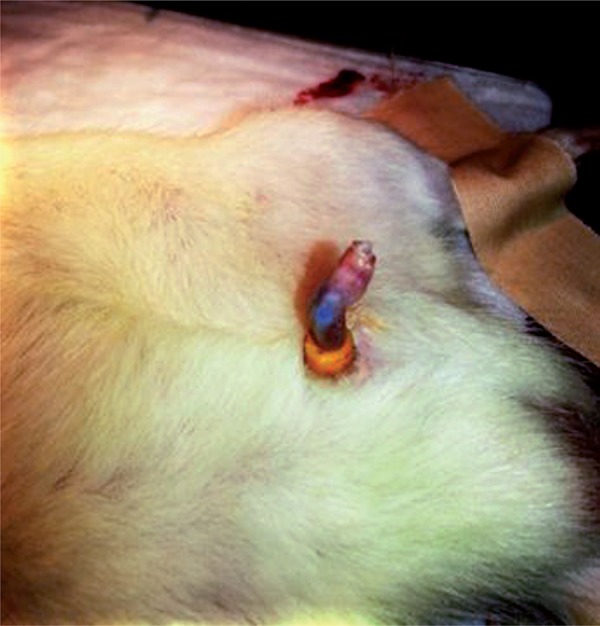
Priapism model induced in the rat penis using a constrictor band.

Anesthesia in all surgical procedures was established with 80 mg/kg ketamine and 10 mg/kg xylazine. The priapism model was induced using the vacuum and constrictor band previously described in the literature (14). A 50 cc irrigation injector was attached to the base of the flaccid rat penis and used like a vacuum device. A constriction band, which was cut from a 16 Fr foley catheter in 2 mm slices, was attached to the tip of the vacuum erection device before vacuum was applied to the penis. Erection was induced by placing the tip of the syringe to the base of the rat penis and withdrawing it to create a 20 cc negative pressure. Once sufficient erection had been induced, the constriction band was removed from the syringe and attached to the base of the penis (Figure-1). The powdered dipyridamole (Koçak Farma, Turkey) used in the experiment was dissolved in DMSO (dimethyl sulfoxide) at 10mg/ml. This was injected intraperitoneally at a dose of 10 mg/kg into rats weighing 400-450 gr. Drug dose was determined based on the report demonstrated that dipyridamole protects rat testis against testicular I/R injury (11).

**Control Group:** Three-milliliter blood specimens were collected from the vena cava inferior (VCI) in order to determine basal serum malondialdehyde (MDA), serum ischemia modified albumin (IMA), serum total antioxidant status (TAS), serum total oxidant status (TOS) and serum oxidative stress index (OSI) values. Penectomy was performed and tissues were placed in a 10% formaldehyde solution for basal histopathological examination of penile tissues.

**Priapism/Reperfusion (P/R) Group:** Thirty minute reperfusion was established by removing the constrictor band on the penis following 1 h priapism.

**Figure 2:**
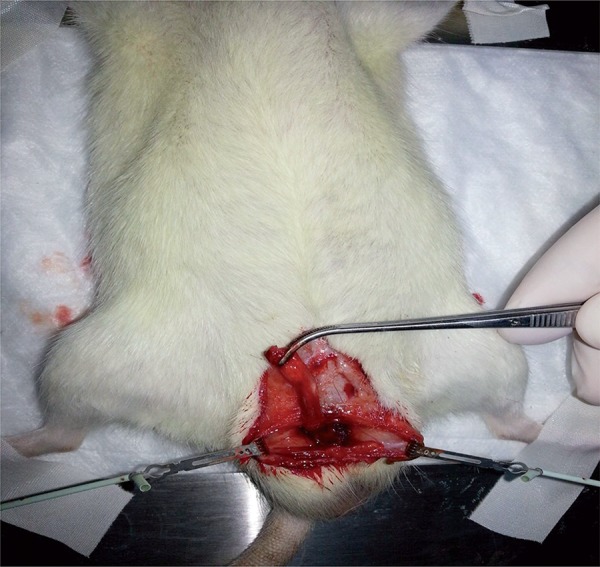
Dissected rat penis.

**Priapism/Reperfusion+DMSO (P/R+DMSO) Group:** The same surgical procedure was performed as in the P/R group. This group received the same volume of DMSO solution as the P/R+D group without dipyridamole intraperitoneally, ½ h before reperfusion (Figure-2).

**Priapism/Reperfusion+Dipyridamole (P/R+D) Group:** The same surgical procedure was performed as in the P/R group. Half an hour before reperfusion, 10 mg/kg dipyridamole were administered intraperitoneally.

Following 30 min reperfusion, 3ml blood specimens were collected from all animals in order to determine serum MDA, IMA, TAS, TOS and OSI values. Penectomy was performed on all animals at the end of the experiment and penises were placed in 10% formaldehyde solution for histopathological examination of penile tissues.

### Histopathological analysis

Penis tissue was extracted from rats from all groups at the end of the study. Penis tissues, taken from approximately the same areas, were kept for 72 h in 10% formaldehyde solution for histopathological examination. Tissue samples were passed through 70%, 90%, 96% and 100% alcohol series for dehydration. They were then passed through xylene solution. Tissues were fixed in paraffin blocks and sections 5 µm in thickness were taken using a fully automated microtome. Following deparaffinization, sections were stained with Masson’s trichrome for detailed histological examination and for better investigation of the cavernous structure (15).

Preparates were evaluated by an experienced and blinded histologist using a light microscope (Olympus BX-51, Olympus Optical Co., Tokyo, Japan). General histological architecture was evaluated. Pathological findings (tunica albuginea tear, endothelial injury) were evaluated semi-quantitatively on a scale of 0 to 3 (0: None, 1: Mild, 2: Moderate, 3: Severe). Groups were also examined using a light microscope at a magnification of 200X on Analysis 5 Research (Olympus Soft Imaging Solutions, Münster, Germany) software for measurement of cavernous tissue sinusoidal area. Means were calculated for sinusoidal area measurements in five different areas for each group.

**Masson’s Trichrome Staining:** Following deparaffinization, samples were kept in bovine solution in an oven at 56ºC for 1 h. Samples were then kept until they turned yellow. After being kept in water for 2 min they were again washed in water. They were then kept for 5 min in combined trichrome dye. They were then again washed in water, and finally dried after being passed through alcohol.

## BIOCHEMICAL ANALYSIS

### Serum MDA Activity Assay

The method described by Yagi was used to determine lipid peroxidation in rat serum samples in the form of MDA concentration (16). Tetramethoxypropane was employed as a standard, and MDA levels were expressed as nmol/mL.

### Measurement of Ischemia-Modified Albumin (IMA)

Assessment of reduced cobalt to albumin binding capacity (IMA level) was performed based on the colorimetric method described by Bar-Or et al. (17). Briefly, 200 µL of rat serum was added to 50 µL of 0.1% cobalt chloride (Sigma, CoCl_2_.6H_2_O) in H_2_O. The solution was gently shaken and then left for 10 minutes to ensure sufficient cobalt albumin binding. Fifty microliters of dithiothreitol (DTT) (Sigma, USA 1.5 mg/ml H_2_O) was added as a colorizing agent. After 2 min the reaction was quenched with the addition of 1.0 mL of 0.9% NaCl. Colorimetric control specimens were prepared for preoperative and postoperative serum samples with 50 µL of distilled water replacing 50 µL of 1.5 mg/mL DTT. Specimen absorbencies were analyzed at 470 nm on a spectrophotometer (Shimadzu UV1601, Ausburn, Australia). The color of the specimens with DTT was compared against the colorimetric control tubes. The results were expressed as absorbance units (ABSUs).

### Measurement of Total Oxidant Status (TOS)

TOS levels were determined using a method previously described by Erel (18). Serum TOS levels were calculated in µmol H_2_O_2_ equivalent/L.

### Measurement of total antioxidant status (TAS)

Total antioxidant status levels were calculated based on the method described by Erel (19) and expressed as mmol Trolox equivalent/L.

### Calculation of oxidative stress index (OSI)

OSI was calculated as the TOS: TAS ratio. TAS units in the form of mmol Trolox equivalent/L were converted to µmol Trolox equivalent/L, with OSI being determined using the formula OSI=[(TOS, µmol H_2_O_2_ equivalent/L)/(TAS, µmol Trolox equivalent/L)x100] (20).

### Statistical analysis

Kruskal-Wallis analysis of variance (Bonferroni-corrected Mann Whitney U test) was used to compare the groups. P<0.05 was regarded as statistically significant.

## RESULTS

### Histopathological Findings

Common parameters in all tissues, sinusoidal area (µm^2^), tears in the tunica albuginea and injury in the sinusoidal endothelium were investigated. Assessment was performed semi-quantitatively, with findings being scored between 0 and 3 (0: None, 1: Mild, 2: Moderate, 3: Severe). Distribution of histopathological parameters in all groups is shown in Table-1. No significant difference was determined between the groups in terms of sinusoidal area (µm^2^) (p>0.05). Significantly more tears in the tunica albuginea were determined in the P/R group compared to the control group (p<0.05). Comparing the P/R+D and P/R groups, dipyridamole resulted in significantly fewer tears in the tunica albuginea (p<0.05). In terms of endothelial injury, significantly more damage was determined in the P/R group compared to the control group (p<0.05). Comparing the P/R+D and P/R groups, dipyridamole caused a decrease in endothelial injury, but the difference was not statistically significant (p>0.05).

**Table 1:** Groups’ histological data.

Groups	Control	P/R	P/R+DMSO	P/R+D	p
Sinusoidal area (µm^2^)	3550±1096	4959±1187	4349±600.6	3550±393.9	0.0312
Tunica albuginea tear	0.3333±0.5164	2.5±0.5477	0.8333±0.4082	1.167±0.4082	0.0005
Endothelial injury	0.1667±0.4082	2.333±0.5164	0.5±0.5477	1.5±0.5477	0.0005

**Figure 3:**
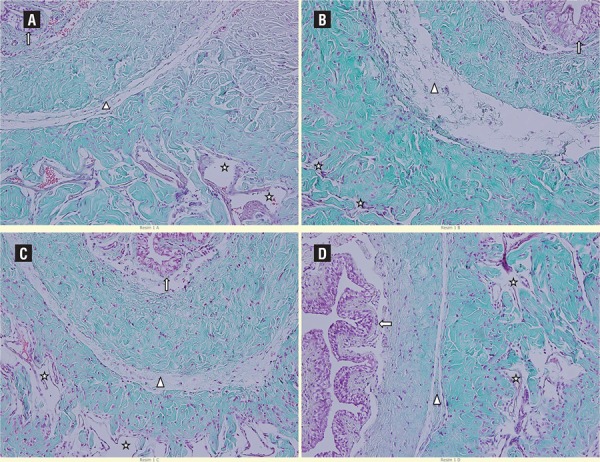
Histopathological examination of the penis from each experimental group (Masson’s trichrome staining x200). A: Group 1, B: Group 2, C: Group 3, D: Group 4.

A normal corpus cavernosum and corpus spongiosum appearance was observed in the control group. Normal morphology was also observed in the surrounding tunica albuginea and the external surrounding superficial fascia. Histological data for vascular spaces and the surrounding smooth muscle layer were normal (Figure-3A). In the P/R group, tears in the tunica albuginea surrounding the corpus spongiosum and connective tissue irregularities were present. The urethral epithelium had a normal appearance. Damage was observed in the endothelial cells lining vascular spaces in the corpus cavernosum. Sinusoidal areas had decreased in size (Figure-3B). In the P/R+DMSO group, a normal appearance was observed in the urethral structure and the tunica albuginea around the corpus spongiosum and corpus cavernosum. The vascular endothelium around the corpus cavernosum had a normal morphology (Figure-3C). In the P/R+D group, the tunica albuginea surrounding the corpus spongiosum and urethral epithelium had a normal appearance. Although occasional damage was observed in the endothelial cells around the vascular spaces in the corpus spongiosum, a close to normal appearance was observed in most of the area (Figure-3D).

**A)** Urethral epithelium (↑), tunica albuginea (∆), sinusoidal area (star); **B)** Urethral epithelium (↑), tunica albuginea (∆), sinusoidal area (star); **C)** Urethral epithelium (↑), tunica albuginea (∆), sinusoidal area (star); **D)** Urethral epithelium (↑), tunica albuginea (∆), sinusoidal area (star).

### Biochemical Findings

Distribution of biochemical parameters in all groups is shown in Table-2. Although an increase was observed in MDA values in the P/R group, no statistically significant difference in MDA was observed among the groups (p>0.05). Although an increase in serum IMA levels was observed in rats in the P/R group, no significant improvement in IMA levels was observed in the P/R+D group (p>0.05). A significant increased in TOS values was observed in the P/R+D group compared to the P/R group (p<0.05). A significant increase in terms of TAS was observed in the P/R group compared to the control group (p<0.05), while a decrease was observed in the P/R+D group compared to the P/R group (p>0.05). OSI values were similar in the control and P/R groups (p>0.05), while an increase was observed in the P/R+D group compared to the P/R group (p<0.05).

**Table 2:** Groups’ biochemical data.

Groups	Control	P/R	P/R+DMSO	P/R+D	p
TOS (µmoIH_2_O_2_Eq/L)	11.06±2.224	10.08±3.029	22.06±7.188	19.44±4.715	0.0008
TAS (mmol/L)	2.404±0.1754	2.808±0.140	2.748±0.161	2.495±0.209	0.0075
OSI	0.4625±0.102	0.358±0.104	0.795±0.226	0.7918±0.233	0.0012
Serum MDA (nmol/L)	0.3087±0.044	0.3865±0.088	0.3515±0.089	0.3803±0.061	0.2245
IMA (ABSU/gr)	0.7398±0.051	0.7578±0.011	0.8015±0.036	0.7315±0.044	0.0452

## DISCUSSION

Priapism may be defined as a prolonged erection due to disturbance of the mechanisms that control penile tumescence, rigidity and flaccidity (21). Ischemic priapism refers to a painful and continuing rigid erection characterized by little or no cavernous blood flow. Hypoxia, hypercarbia and acidosis develops in cavernous blood gas (22).

Ultrastructural changes in smooth muscle occur after 12 h in ischemic priapism, focal necrosis after 24 hs and finally necrosis and transformation of fibroblast-like cells after 48 h. If priapism is untreated or treated late (>24 hs) necrosis in cavernous smooth muscle, irreversible corporal fibrosis and permanent erectile dysfunction occur (4). The most significant determining factor in the prevention of tissue damage after ischemic priapism is duration of ischemia, which is directly correlated with reperfusion injury. Several studies have investigated I/R injury in different tissues (6, 11, 23). The purpose of this study was to investigate early histological and biochemical changes and the probable protective effects against I/R injury of dipyridamole before the emergence of irreversible damage following 1 h acute ischemic priapism in a model of ischemic priapism experimentally induced in rats.

Dipyridamole is a platelet inhibitor that increases cAMP levels by inhibiting the enzyme phosphodiesterase in platelets. It blocks reabsorption of adenosine by cells and increases interstitial adenosine concentrations (24, 25). It causes vasodilation by increasing adenosine formation, and probably improves tissue perfusion through a combination of its antiplatelet and vasodilator effects (10). Various studies have investigated the effects of dipyridamole on I/R injury. In a study of the antioxidant properties of dipyridamole, Vargas et al. reported that dipyridamole scavenges reactive oxygen radicals (ROS) released by human polymorphonuclear leukocytes (26). Iuliano et al. reported that dipyridamole possesses antioxidant properties (27). An experimental study determined that dipyridamole protects liver cells against warm I/R injury (28). Karagüzel et al. reported that the use of dipyridamole before testicular reperfusion in testicular torsion protects the testis against the long-term effects of I/R injury (11). The intraperitoneal 10 mg/kg dose of dipyridamole used in that study of testicular torsion was also employed in our priapism model which is another model of I/R injury. It may be possible for further studies to use different doses of dipyridamole to show increased effectiveness.

Very few experimental drug studies have investigated protective efficacy against I/R injury occurring in priapism (14, 29). Ours is the first study in the literature to investigate the effect of dipyridamole in an experimental priapism model. Although dipyridamole is a molecule that is safely used in various indications because of its anti-aggregating properties in humans, there are no studies concerning its use in humans with clinical priapism. There is a strong probability that as a result of this and future studies, dipyridamole will be used to prevent potential I/R injury in priapism, a new indication in humans, through investigation of the most appropriate and effective dosage.

Although histopathological evaluation of tunica albuginea and endothelial injury parameters in this study revealed ischemic changes, no change in favor of ischemia was determined in the levels of serum IMA and MDA and other biochemical parameters. Although a decrease was determined in TAS and IMA values in the group receiving dipyridamole compared to the P/R group, this was not statistically significant. Biochemical data not exhibiting significant differences in ischemic priapism, a compartment syndrome analogue, may be attributed to the 30 min reperfusion time we used being too short for ischemic tissue products to enter the circulation with reperfusion and establish a systemic effect. A longer reperfusion process is needed for penile ischemic products to exhibit systemic effects and for significant changes to be identified in ischemic parameters in serum. This study represents the first investigation in a priapism model of IMA, a biochemical parameter that has been investigated and found to be of value in such different models of I/R injury as testicular torsion (30, 31).

The effects of I/R injury occurring in priapism and the probable protective effects of dipyridamole were investigated through histological examination of the sinusoidal area, tears in the tunica albuginea and damage parameters in the sinusoidal endothelium. Examination of I/R injury in the P/R group revealed tears in the tunica albuginea surrounding the penis corpus spongiosum and irregularities in connective tissue, injury to the endothelial cells around the vascular spaces in the penis corpus cavernosum and contractions in sinusoidal areas. Examination of the penises of rats given dipyridamole revealed improvement in tunica albuginea and endothelial injury and showed that dipyridamole, which has antioxidant and anti-inflammatory properties, exhibits histopathological protective effects in I/R injury caused by priapism.

A certain amount of I/R injury will develop in penis corpus cavernosum tissue in patients with priapism due to various etiological causes, even if treated in the early period. Further studies are needed on the subject of the clinical outcomes that I/R may cause in the long-term. This study represents one of the rare examples of medical therapies that can be used in order to reduce I/R injury that may occur in patients treated in the early period.

The main limitation of this study is that the length of ischemia in the model of priapism used was limited to 1 hour. More significant results in terms of biochemical data may be obtained with a longer duration of ischemia. Another limitation is the difficulty in applying the animal model used in this study to humans and in determining appropriate dose selection.

## CONCLUSIONS

In conclusion, this experimental study shows that the application of dipyridamole before reperfusion in a model of ischemic priapism has potential protective effects against histopathological injury that may develop in the penis. Further and more comprehensive studies are now needed in order to prevent I/R injury in ischemic priapism.
